# Association of perioperative initiation of gabapentin versus pregabalin with kidney function: a target trial emulation study

**DOI:** 10.3389/fmed.2024.1488773

**Published:** 2024-12-10

**Authors:** Yanfang He, Liqian Mo, Juan Li, Dongying Lu, Jinlei Niu, Ying Li, Qiying Zeng, Yueming Gao

**Affiliations:** ^1^Department of Pediatrics, Nanfang Hospital, Southern Medical University, Guangzhou, China; ^2^Department of Pharmacy, Nanfang Hospital, Southern Medical University, Guangzhou, China; ^3^Department of Nephrology, Peking University Third Hospital, Beijing, China

**Keywords:** gabapentin, pregabalin, perioperative analgesia, kidney function, acute kidney injury

## Abstract

**Background:**

Gabapentinoids, such as gabapentin and pregabalin, are opioid substitutes commonly included in perioperative multimodal analgesia regimens. We investigated whether the initiation of gabapentin and pregabalin during the perioperative period have varying effects on the adverse renal outcomes.

**Methods:**

This study included adult participants who received surgery in the INSPIRE database. The exposure of interest was the initiation of pregabalin or gabapentin during the perioperative period. The primary outcome was renal function decline. Secondary outcomes included incident chronic kidney disease (CKD), hospital-acquired acute kidney injury (AKI), and in-hospital mortality. We conducted a propensity score to balance the baseline characteristics. Cox proportional hazard regression was used to estimate the hazard ratio (HR) of the initiation of gabapentin compared with pregabalin.

**Results:**

Among 640 pairs of pregabalin and gabapentin initiators in the matched cohort, the initiation of gabapentin was associated with a higher risk of kidney function decline (HR, 1.40; 95% confidence interval [CI], 1.04–1.89) as compared with pregabalin. After excluding participants who were diagnosed with CKD at the baseline, the initiation of gabapentin was associated with a higher risk of incident CKD (HR, 1.46; 95% CI, 1.03–2.05) as compared with pregabalin. For the in-hospital outcomes, the proportion of AKI and mortality were similar between participants initiating gabapentin and pregabalin. In addition, the risk of kidney function decline did not vary across each subgroup.

**Conclusion:**

The initiation of gabapentin during the perioperative period was associated with a higher risk of kidney function decline and incident CKD as compared with pregabalin.

## 1 Introduction

Adequate postoperative pain control is an important component of the Enhanced Postoperative Recovery (ERAS) pathway, which has been related to better outcomes, shorter hospital stays, and lower costs (1–3). In recent years, multimodal analgesia targeting various pain pathways has been an increasingly adopted strategy in the ERAS pathway, which aims to improve postoperative pain relief with minimal or no opioid consumption, and thus reduce opioid-related adverse events (4). Gabapentinoids, such as pregabalin and its predecessor, gabapentin, are now commonly included in multimodal analgesia regimens to reduce postoperative pain and opioid requirements, which were also found to reduce the incidence of postoperative nausea, vomiting, and pruritus (5, 6). However, despite these benefits, gabapentinoids have been reported to increase the risk of adverse effects such as sedation, dizziness, visual disturbances, ataxia, cognitive impairment, and respiratory depression, particularly when used concurrently with opioids (6–8).

Gabapentinoids were originally designed as analogs of the inhibitory neurotransmitter gamma-aminobutyric acid (GABA), but they have no significant antagonistic effect on GABA_A_ or GABA_B_ receptors (9). Instead, their mechanism of action involves targeting the α-2-δ subunit of presynaptic voltage-dependent calcium channels in the spinal cord and peripheral nerves, inhibiting calcium influx, and thus decreasing the release of excitatory neurotransmitters and reducing spinal sensitization (10). Gabapentinoids are not metabolized by the liver; they are primarily eliminated by the kidneys in an unchanged form, with clearance proportional to the creatinine clearance. Accumulation of gabapentinoids can lead to kidney failure and other adverse effects (11). Therefore, in patients with chronic kidney disease (CKD), appropriate dosing of gabapentinoids is crucial to minimize the risk of adverse events (12). A single center retrospective cohort study reported that patients with decreased creatinine clearance (<60 mL/min) often take inappropriate high-dose gabapentin, which may exacerbate adverse effects (13). Even in patients whose kidney function was previously normal, several case reports indicated that gabapentin can directly induce rhabdomyolysis and cause acute kidney injury, suggesting potential renal adverse reactions of gabapentin (14–19).

Although gabapentin and pregabalin share similar chemical structures and mechanisms of action but differ considerably in pharmacodynamic and pharmacokinetic profiles (11). Pregabalin, which developed after gabapentin, is more potent and has the benefits of more rapid peak blood concentration and better bioavailability than gabapentin (20). Previous studies have reported that substituting gabapentin with pregabalin may result in improved pain relief and fewer adverse events, such as sedation, dizziness, and peripheral edema (21). However, to our knowledge, it is uncertain whether the risk of adverse kidney outcomes varies between gabapentin and pregabalin, as there are no direct comparisons.

Therefore, this study aims to compare the risk of adverse kidney outcomes in participants undergoing perioperative initiating gabapentin or pregabalin, hoping to provide some reference for the selection of Gabapentinoids during the perioperative period.

## 2 Methods

### 2.1 Target trial emulation

We emulated a target trial with new-user, active comparator design to compare the risk of kidney outcomes in participants underwent surgery and initiated pregabalin or gabapentin during the perioperative period. Supplementary Table S1 summarizes the key design elements of this trial.

### 2.2 Data source

Participants were identified from a publicly available research dataset in perioperative medicine, which includes appropriately 130,000 cases who underwent anesthesia for surgery at an academic institution in South Korea between January 2011 and December 2020. This comprehensive dataset includes patient characteristics such as age, sex, American Society of Anesthesiologists physical status classification, diagnosis, surgical procedure code, department, and type of anesthesia. It also includes vital signs in the operating theater, general wards, and intensive care units (ICUs), laboratory results from 6 months before admission to 6 months after discharge, and medication during hospitalization. Complications include total hospital and ICU length of stay and in-hospital death. This study followed the Strengthening the Reporting of Observational Studies in Epidemiology (STROBE) reporting guideline.

### 2.3 Study population

**Figure 1:**
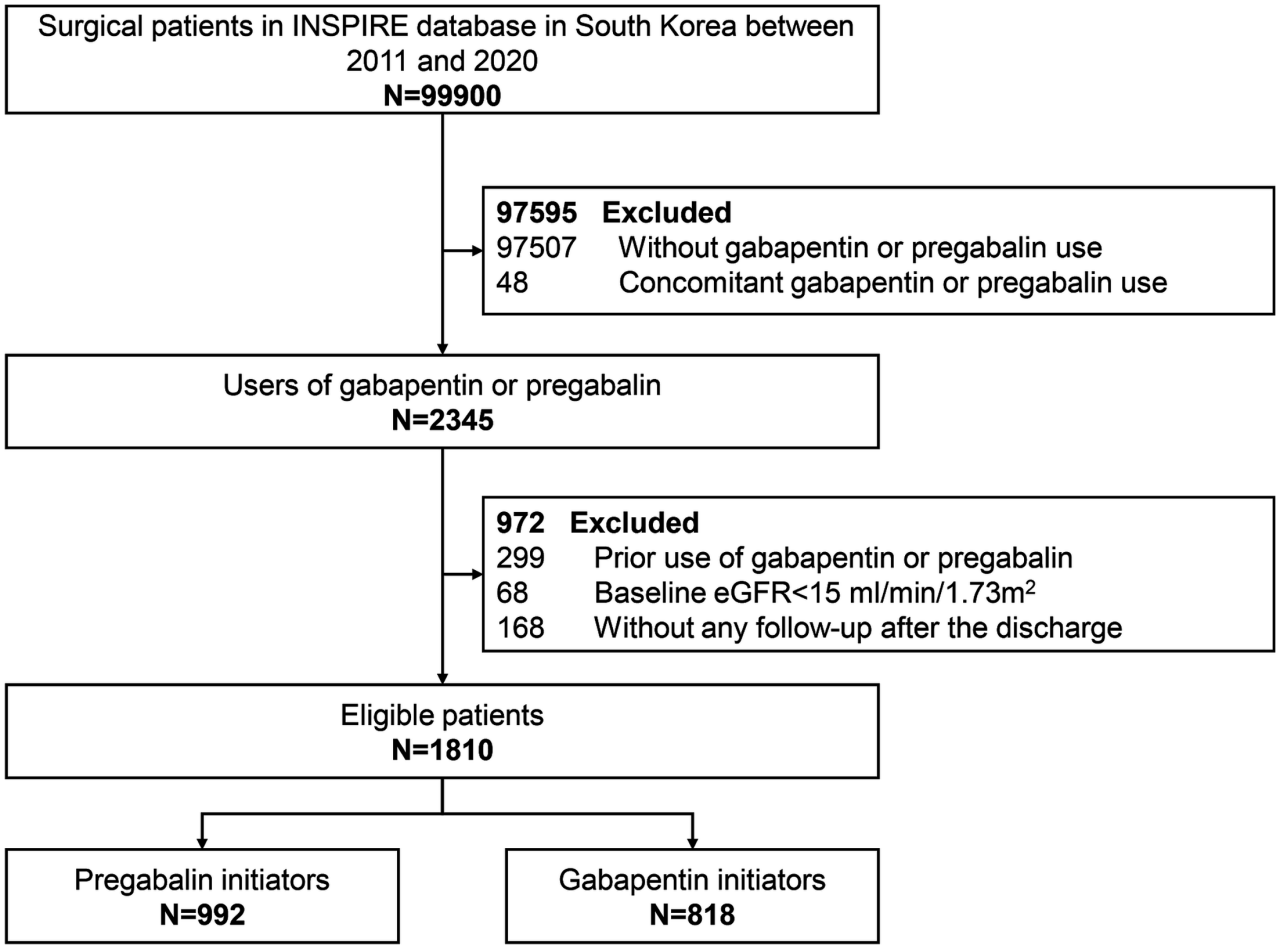
Study flow diagram of participants initiating pregabalin or gabapentin in the INSPIRE database between 2011 and 2020.

This study included participants aged 18–90 years who underwent surgery with general, neuraxial, regional, or monitored anesthesia care, as recorded in the INSPIRE database (22, 23). We included participants who received any prescription of pregabalin or gabapentin during the perioperative period. Participants using both pregabalin and gabapentin concurrently were excluded. Additionally, to avoid prevalent user bias, we excluded participants with prior use of the study drugs. The index date was defined as the date of the first prescription of either pregabalin or gabapentin during the perioperative period. Participants with a baseline estimated glomerular filtration rate (eGFR) <15 mL/min/1.73 m^2^ or those lacking laboratory measurements after discharge were also excluded. The flowchart of participants selection was shown in Figure 1.

### 2.4 Exposure

The exposure of interest was the initiation of pregabalin or gabapentin during the perioperative period, with the start date of treatment for each participant defined as the index date. To mimic the intention-to-treat approach of a randomized clinical trial, participants were considered to remain on the study drug for the entire duration of the analysis.

### 2.5 Outcomes

The primary outcome was the kidney function decline, defined as the >40% decline in eGFR from the baseline within 6 months. Secondary outcomes included the incident CKD within 6 months, hospital-acquired acute kidney injury (HA-AKI), and in-hospital mortality. The incident CKD was defined as the new-onset eGFR < 60 mL/min/1.73 m^2^ and was assessed among participants with baseline eGFR > 60 mL/min/1.73 m^2^. HA-AKI was defined as an increase in serum creatinine (SCr) by 0.3 mg/dL within 48 h or a 50% increase in SCr from the baseline within 7 days according to the KDIGO criteria (24). Methods for determining HA-AKI have been reported in our previous studies (25). Follow-up began at the date of the initiation of study drugs until the occurrence of the outcome of interest, death, or the end of the study period (31 December 2020), whichever came first.

### 2.6 Covariates

The baseline characteristics included the demographic characteristics, body mass index (BMI), calendar year of the drugs initiation, operation and anesthesia-related parameters, intraoperative factors (plasma solution infused and sustained hypotension), diagnosis, vital signs, laboratory results, or prescription and administration of the medications were extracted from the clinical data warehouse of the Seoul National University Hospital (SUPREME version 1.0 and 2.0). Laboratory measurements were recorded from 6 months before the operation to 6 months after the last discharge. Potential confounding factors in our study included age, sex, blood pressure, type of surgery (orthopedic, gastroenterological, cardiac, neurological, and other surgeries), laboratory measurements (eGFR, hemoglobin, and serum albumin), comorbidities (hypertension, diabetes, cancer, heart failure, and coronary heart disease, sepsis), and co-medications (nonsteroidal anti-inflammatory drugs [NASIDs], opioids, renin-angiotensin system inhibitors [RASi], statins, proton pump inhibitors [PPI], aminoglycosides, loop diuretics, and antibiotics).

### 2.7 Statistical analysis

Baseline characteristics for the overall cohort and across the two initiation groups were presented as median (interquartile range, IQR) for continuous variables and frequencies with proportions for categorical variables. Standardized mean differences (SMDs) were computed and presented, with values less than 0.1 considered indicative of balance (26).

To balance baseline characteristics between the two initiation groups, we conducted propensity score matching (PSM) using logistic regression to model the probability of initiating gabapentin, adjusting for baseline covariates described in Table 1. Patients receiving gabapentin were matched with those initiating pregabalin in a 1:1 ratio (nearest-neighbor) based on a maximum caliper width of 0.1 of the standard deviation of the logit of the propensity score. Cumulative incidence curves for kidney function decline and incident CKD were plotted in the matched sample. Incidence rates per 100 person-years with 95% confidence intervals (CIs) were calculated using 1,000 nonparametric bootstrap samples. Cox proportional hazard regression was performed to estimate the hazard ratio of gabapentin initiation compared with pregabalin initiation after PSM, without further adjustment. Our primary analyses adhered to the intention-to-treat principle; thus, participants who initiated pregabalin and subsequently initiated gabapentin were retained in the pregabalin group, and vice versa.

**Table 1:** Baseline characteristics between participants with pregabalin or gabapentin initiation before and after PSM.

Characteristics	Before PSM				After PSM		
	Overall (*N* = 1,810)	Pregabalin (*N* = 992)	Gabapentin (*N* = 818)	SMD	Pregabalin (*N* = 622)	Gabapentin (*N* = 622)	SMD
Age, yr	65 (55–70)	65 (55–75)	60 (50–70)	0.287	65 (55–70)	65 (50–70)	0.009
Sex, male	1,029 (56.9)	589 (59.4)	440 (53.8)	0.113	346 (55.6)	348 (55.9)	0.006
SBP, mmHg	118 (102.5–141)	118 (102.4–140.1)	118.5 (103–142)	0.027	118 (102–143)	118.8 (103–142.4)	0.029
DBP, mmHg	69 (60–80.8)	69 (60–80)	70 (60–82)	0.089	70 (60.1–81)	71 (60–81.4)	0.025
BMI	24 (21.4–26.5)	24.4 (21.9–27)	23.4 (20.9–26)	0.040	24.1 (21.6–26.8)	23.6 (21.1–26.2)	0.029
Emergency surgery, %	288 (15.9)	150 (15.1)	138 (16.9)	0.048	114 (18.3)	111 (17.8)	0.013
Trauma, %	221 (12.2)	84 (8.5)	137 (16.7)	0.251	72 (11.6)	92 (14.8)	0.095
Cardiopulmonary bypass, %	133 (7.3)	101 (10.2)	32 (3.9)	0.247	28 (4.5)	30 (4.8)	0.015
Surgery (%)				0.578			0.030
Orthopedic	711 (39.3)	472 (47.6)	239 (29.2)		238 (38.3)	230 (37.0)	
Gastroenterology	110 (6.1)	47 (4.7)	63 (7.7)		45 (7.2)	47 (7.6)	
Cardiac	120 (6.6)	90 (9.1)	30 (3.7)		28 (4.5)	30 (4.8)	
Nervous	194 (10.7)	121 (12.2)	73 (8.9)		67 (10.8)	68 (10.9)	
Other	675 (37.3)	262 (26.4)	413 (50.5)		244 (39.2)	247 (39.7)	
Intraoperative							
Plasma solution infused, ml	500 (0–1,200)	450 (0–1,000)	600 (0–1,500)	0.224	500 (0–1,200)	500 (0–1,300)	0.035
Sustained hypotension, %	872 (48.2)	464 (46.8)	408 (49.9)	0.062	302 (48.6)	300 (48.2)	0.006
Laboratory							
eGFR, mL/min/1.73 m^2^	96.8 (77.7–110.7)	96.6 (75.3–108.9)	97.4 (79.4–112.4)	0.129	97.6 (75.4–111.4)	96 (78.4–111.6)	<0.001
Hemoglobin, g/L	11 (9.8–12.2)	11 (9.8–12.2)	11 (9.8–12.2)	0.004	10.9 (9.7–12.3)	11 (9.8–12.3)	0.021
Serum Albumin, g/dl	3.5 (3.2–3.8)	3.5 (3.2–3.8)	3.5 (3.1–3.8)	0.160	3.5 (3.1–3.8)	3.5 (3.1–3.8)	0.012
Comorbidities (%)							
Hypertension	173 (9.6)	100 (10.1)	73 (8.9)	0.039	55 (8.8)	54 (8.7)	0.006
Diabetes	204 (11.3)	127 (12.8)	77 (9.4)	0.108	70 (11.3)	65 (10.5)	0.026
Cancer	655 (36.2)	269 (27.1)	386 (47.2)	0.425	240 (38.6)	243 (39.1)	0.010
Sepsis	9 (0.5)	6 (0.6)	3 (0.4)	0.034	4 (0.6)	2 (0.3)	0.046
Heart failure	38 (2.1)	21 (2.1)	17 (2.1)	0.003	14 (2.3)	11 (1.8)	0.034
Hepatic failure	4 (0.2)	3 (0.3)	1 (0.1)	0.039	3 (0.5)	1 (0.2)	0.057
Coronary heart disease	103 (5.7)	70 (7.1)	33 (4.0)	0.132	29 (4.7)	31 (5.0)	0.015
Medications (%)							
NSAIDs	601 (33.2)	360 (36.3)	241 (29.5)	0.146	188 (30.2)	192 (30.9)	0.014
Opioids	1,227 (67.8)	680 (68.5)	547 (66.9)	0.036	411 (66.1)	414 (66.6)	0.010
Loop diuretics	170 (9.4)	120 (12.1)	50 (6.1)	0.209	48 (7.7)	46 (7.4)	0.012
RASi	211 (11.7)	124 (12.5)	87 (10.6)	0.058	75 (12.1)	72 (11.6)	0.015
Statin	420 (23.2)	274 (27.6)	146 (17.8)	0.235	129 (20.7)	131 (21.1)	0.008
Aminoglycosides	17 (0.9)	11 (1.1)	6 (0.7)	0.039	5 (0.8)	6 (1.0)	0.017
PPI	421 (23.3)	264 (26.6)	157 (19.2)	0.177	130 (20.9)	135 (21.7)	0.020
Antibiotics	1,055 (58.3)	570 (57.5)	485 (59.3)	0.037	347 (55.8)	351 (56.4)	0.013
In-hospital outcomes (%)^*^							
AKI	41 (2.3)	12 (1.2)	29 (3.5)	0.154	11 (1.8)	22 (3.5)	0.110
Mortality	28 (1.5)	21 (2.1)	7 (0.9)	0.104	11 (1.8)	5 (0.8)	0.086

* In-hospital outcomes were not including in the propensity score model.

AKI, acute kidney injury; BMI, body mass index; DBP, diastolic blood pressure; eGFR, estimated glomerular filtration rate; NSAIDs, Non-Steroidal Anti-inflammatory Drugs; PPI, proton pump inhibitors; PSM, propensity score matching; RASi, renin-angiotensin system inhibitors; SBP, systolic bolld pressure; SMD, standardized mean difference.

### 2.8 Sensitivity and subgroup analyses

Four sensitivity analyses were conducted. First, we used propensity score overlap weighting instead of matching and repeated our analysis. Second, participants with less than 90 days of follow-up were excluded to minimize the potential for reverse causality. Third, participants who AKI or died during hospitalization were excluded to mitigate the impact of severe conditions on prognosis. Fourth, in addition to the intention-to-treat analysis, we performed a sensitivity analysis using a per-protocol approach. In the per-protocol analysis, participants were censored at the date they switched study drugs (deviated from the initially initiated drug). Fifth, we re-define the kidney outcome as a sustained decrease in eGFR >40% from baseline, confirmed by two consecutive SCR measurements. Sixth, we performed the same analysis under the assumption of Missing Non at Random (MNAR) mechanism and compared these results with the primary analysis. Subgroup analyses were conducted to explore potential effect modifications among participants stratified by age (≥60 and <60 years), sex, hypertension, diabetes, cancer, and use of statins and PPI. Missing values were imputed using multiple imputation (using the ‘mice’ package in R).

## 3 Results

### 3.1 Baseline characteristics

Data from 1,810 eligible participants were included in this analysis (median (IQR) age, 65 [55–70] years; 1,029 [56.9%] male; and 711 [39.3%] underwent orthopedics surgery; with median [IQR] eGFR, 96.8 [77.7–110.7]). Participants initiating pregabalin and gabapentin differed in pertinent baseline characteristics (defined as SMDs >10%). For instance, gabapentin initiators were younger, had a lower proportion undergoing orthopedic surgery, were more likely to have a diagnosis of cancer, and were less likely to use statins and PPI compared to those initiating pregabalin. After PSM, 640 pregabalin initiators were matched to 640 gabapentin initiators. All variables included in the propensity score model were balance between the two groups (no SMDs exceeded 0.1). Regarding in-hospital outcomes, the proportions of AKI and mortality were similar between participants initiating gabapentin and pregabalin. During the 10-year study, there was no notable trend in the initiation rates of gabapentin versus pregabalin (Supplementary Figure S1). Baseline characteristics between participants with pregabalin or gabapentin initiation before and after PSM were shown in Table 1. The proportion of missingness of covariates were shown at Supplementary Table S2.

### 3.2 Risk of kidney function decline and incident CKD

**Figure 2:**
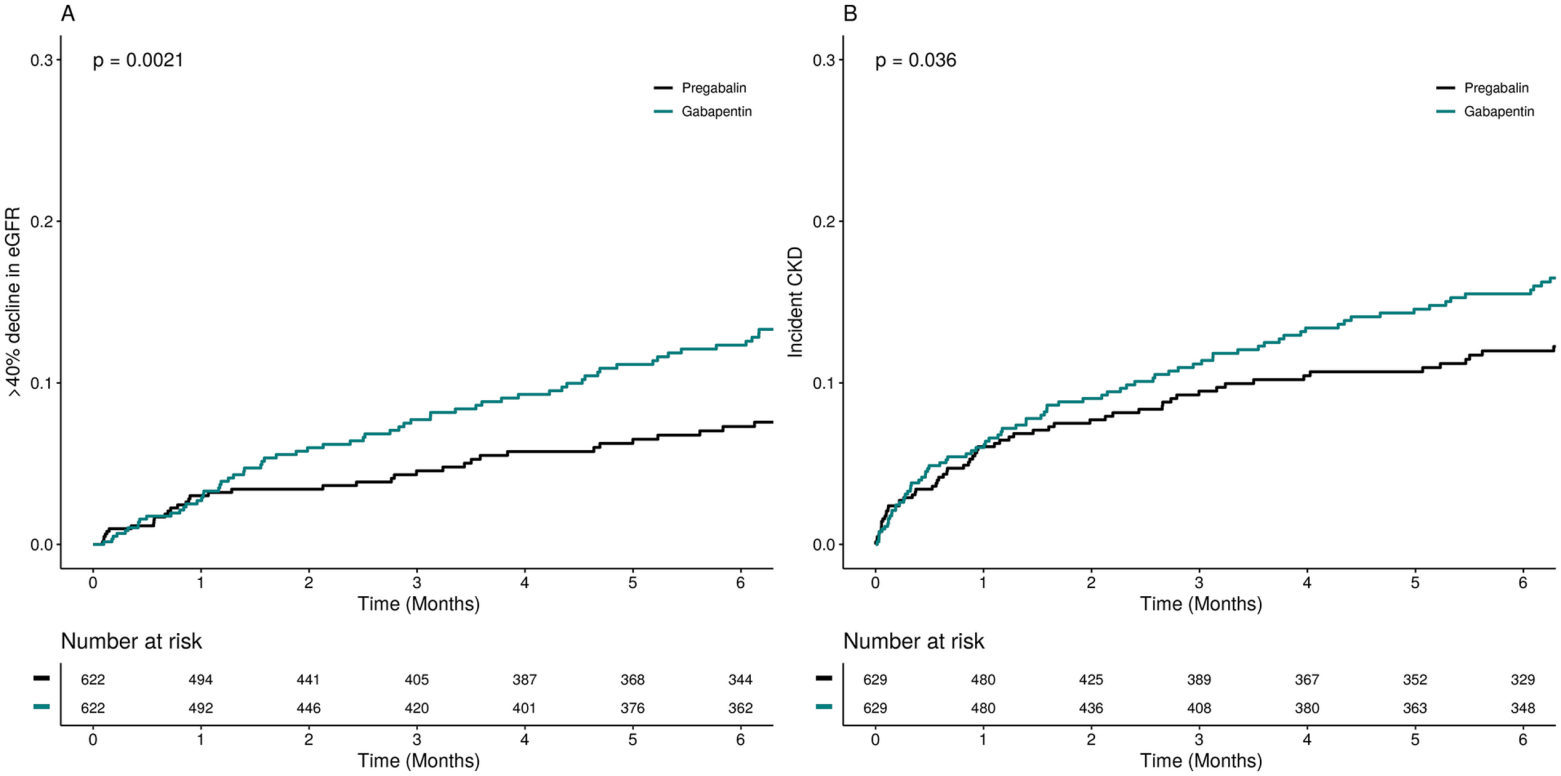
Cumulative incidence of the kidney outcomes among pregabalin initiators and gabapentin initiators.

**Figure 3:**
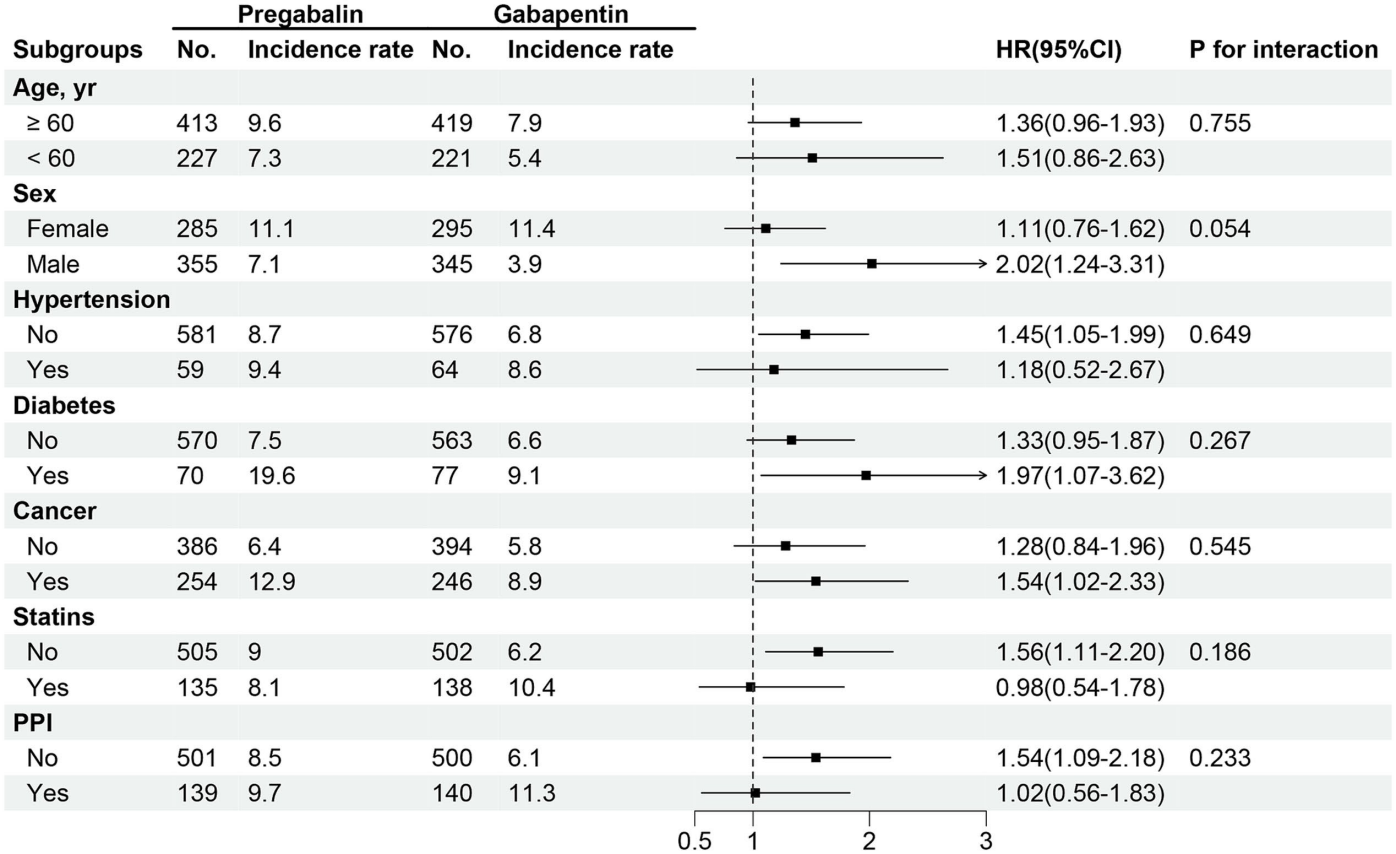
HRs for the association between pregabalin initiators versus gabapentin initiators and risk of kidney function decline among different subgroups.

In the propensity score-matched sample participants (Table 2), the frequency of the SCR measurements after the study drugs initiation was consistent between the two groups (Supplementary Figure S2). The incidence rate of kidney function decline per 100 person-year of follow-up was 8.20 (6.63–10.19) among pregabalin initiators and 9.70 (8.16–11.49) among gabapentin initiators. Gabapentin initiation was associated with a higher risk of kidney function decline (HR 1.40; 95% CI 1.04–1.89) compared to pregabalin (Figure 2 and Table 2). Subgroup analysis indicated no significant variation in the risk of kidney function decline across different subgroups (Figure 3, *p* for interaction >0.05).

**Table 2:** The association of kidney outcomes with the initiation of gabapentin versus pregabalin after PSM.

Kidney outcomes	No.	Event	Person-year	Incidence rate (95% CI)^*^	HR (95% CI)	sHR (95% CI)
Kidney function decline						
Pregabalin	622	77	989	7.79 (6.23–9.68)	1.00 (Reference)	
Gabapentin	622	125	1,248	10.01 (8.43–11.85)	1.6 (1.18–2.17)	1.61 (1.19–2.18)
Incident CKD^#^						
Pregabalin	629	106	966	10.98 (9.11–13.16)	1.00 (Reference)	
Gabapentin	629	149	1,152	12.93 (11.08–15.04)	1.33 (1.02–1.75)	1.33 (1.02–1.74)

* Per 100 person-year.

# Incident CKD was assessed among participants with baseline eGFR > 60 mL/min/1.73 m^2^.

PSM, propensity score matching; CKD, chronic kidney disease; HR, hazard ratio; sHR, sub-distribution hazard ratio.

To assess the association between gabapentin and incident chronic kidney disease (CKD) (Table 2), we excluded 644 participants diagnosed with CKD at baseline and performed separate PSM in the incident CKD cohort. The incidence rate of CKD was 7.88 per 100 person-years among pregabalin initiators and 10.21 per 100 person-years among gabapentin initiators, indicating a significantly higher risk of incident CKD associated with gabapentin initiation (HR 1.46; 95% CI 1.03–2.05).

### 3.3 Sensitivity analyses

Similar findings were observed in sensitivity analyses using propensity score overlap weighting (Supplementary Table S3). After excluding participants who experienced outcomes or were lost to follow-up within 90 days (Supplementary Table S4), initiation of gabapentin was significantly associated with an increased risk of kidney function decline (HR 1.47; 95% CI 1.06–2.02) and incident chronic kidney disease (HR 1.47; 95% CI 1.03–2.10) compared to pregabalin. Consistent results were found in sensitivity analyses after excluding participants who developed acute kidney injury or died during hospitalization (Supplementary Table S5). In the per-protocol analysis, increased risks of kidney function decline and incident chronic kidney disease were observed among participants initiating gabapentin compared to those initiating pregabalin (Supplementary Table S6). Consistent results were obtained when re-defined the kidney outcome to require confirmation by two consecutive eGFR measurements (Supplementary Table S7) and assuming a MNAR mechanism (Supplementary Table S8).

## 4 Discussion

Using clinical data from participants who underwent surgery under general, neuraxial, regional, and monitored anesthesia care in the INSPIRE database, we conducted a target trial with a new-user, active comparator design to compare the risk of adverse kidney outcomes in participants initiating pregabalin or gabapentin during the perioperative period. We found that initiation of gabapentin was associated with a higher risk of kidney function decline (HR 1.40; 95% CI 1.04–1.89) and incident chronic kidney disease (HR 1.46; 95% CI 1.03–2.05) compared to pregabalin. The results of the sensitivity analyses and subgroup analyses remained consistent. Our study contributes to filling this gap and offers insights that may guide the rational selection of gabapentinoids during the perioperative period.

Gabapentinoids, including gabapentin and pregabalin, are increasingly used in multimodal analgesia regimens to minimize opioid consumption during the perioperative period (5). Administered orally, gabapentinoids are primarily excreted unchanged via the kidneys. Their half-life ranges from 5 to 7 h, which increases with declining kidney function (27). Patients with CKD are particularly susceptible to gabapentin toxicity (28). Therefore, cautious selection of initial doses and dose adjustments are crucial in this patient population (29). A previous population-based cohort study involving 74,084 older adults with CKD examined the 30-day risk of severe adverse events associated with different starting doses of gabapentinoids. The study found that initiating gabapentinoids at higher doses correlated with an increased risk of hospital visits due to encephalopathy, falls, fractures, or hospitalizations for respiratory depression (30). A recent retrospective cohort study focused on older patients during the perioperative period and assessed gabapentin-related adverse effects. PSM revealed that compared to non-users, gabapentin users had a heightened risk of delirium, particularly pronounced in patients with CKD (31). The risk of toxicity is further elevated in patients undergoing dialysis. Research involving hemodialysis patients indicated that higher doses of gabapentin or pregabalin were associated with increased risks of altered mental status, falls, and fractures (32). These findings underscore the importance of judicious use of gabapentinoids based on kidney function, highlighting the need for future research to establish optimal dosing strategies.

Renal functional impairment has been reported as a delayed adverse effect of gabapentin (33). However, the mechanisms by which gabapentin cause renal dysfunction remains poorly understood. Several case reports indicated that gabapentin can directly induce rhabdomyolysis and cause acute kidney injury, even in patients whose kidney function was previously normal (14–19). In addition, in experimental animal models, gabapentin was reported to induce apoptosis and lead to structural alterations in the kidney, including renal tubular epithelial degeneration, hemorrhage, and glomerular atrophy (34). Although pregabalin and gabapentin share similar chemical structures and mechanisms of action, pregabalin is known to be more potent and faster-acting than gabapentin (11). Gabapentin is almost 100% excreted in its original form through the kidneys, which may increase the risk of drug accumulation and toxicity in the kidneys, thereby increasing the risk of kidney damage. In contrast, the proportion of pregabalin excreted through the kidneys is relatively small, which has a relatively small burden on the kidneys (11). Previous research has also suggested that switching from gabapentin to pregabalin could potentially enhance pain relief and reduce adverse events such as sedation, dizziness, and peripheral edema (21). However, the comparative risk of adverse kidney outcomes between gabapentin and pregabalin remains unclear due to the absence of direct comparisons in previous studies. In our study, utilizing a new-user, active comparator design, we found that initiating gabapentin was associated with a higher risk of kidney function decline and incident CKD compared to pregabalin. Importantly, this risk of kidney function decline was consistent across different subgroups analyzed. To our knowledge, our study represents the first direct comparison of kidney adverse outcomes between gabapentin and pregabalin, suggesting that pregabalin may carry a lower risk of kidney adverse events than gabapentin.

The strengths of the current study include its real-world-based dataset, new-user design, and use of hard kidney outcomes. Furthermore, sophisticated statistical methods were employed to mitigate confounding and indication biases. However, the study also has several limitations. Firstly, despite PSM to balance baseline characteristics between gabapentin and pregabalin initiators, residual confounding from unmeasured factors may still impact outcomes. Sensitivity analyses were conducted to address this concern and reinforce result robustness. Secondly, the study did not explore the potential differential impact of varying initial doses of gabapentin or pregabalin on kidney outcomes, warranting future investigations in this area. Thirdly, the study population exclusively comprised patients from South Korea, necessitating validation of findings across diverse populations and geographic regions. Fourthly, due to the scattered distribution of surgical types, we were unable to perform subgroup analyses to access the impact of the heterogeneity in surgical procedures and the unknown intraoperative events. However, we have attempted to adjust for some intraoperative events such as plasma solution infused and sustained hypotension. Lastly, this study is hypothesis-generating and requires further validation through randomized controlled trials.

In conclusion, the initiation of gabapentin during the perioperative period was associated with a higher risk of kidney function decline and incident CKD as compared with pregabalin. These findings suggest that perioperative use of pregabalin might pose a lower risk of adverse kidney outcomes than gabapentin.

## Data Availability

The datasets presented in this study can be found in online repositories. The names of the repository/repositories and accession number(s) can be found at: https://physionet.org/content/inspire/1.3/.
