# Supplementary material for: Association of perioperative initiation of gabapentin versus pregabalin with kidney function: a target trial emulation study
Source: Front Med (Lausanne). 2024 Dec 10;11:1488773. doi: 10.3389/fmed.2024.1488773 (PMC11666351; doi:10.3389/fmed.2024.1488773)
Supplement: Supplementary file 1 [file Data_Sheet_1.docx]

Supplementary Material

**
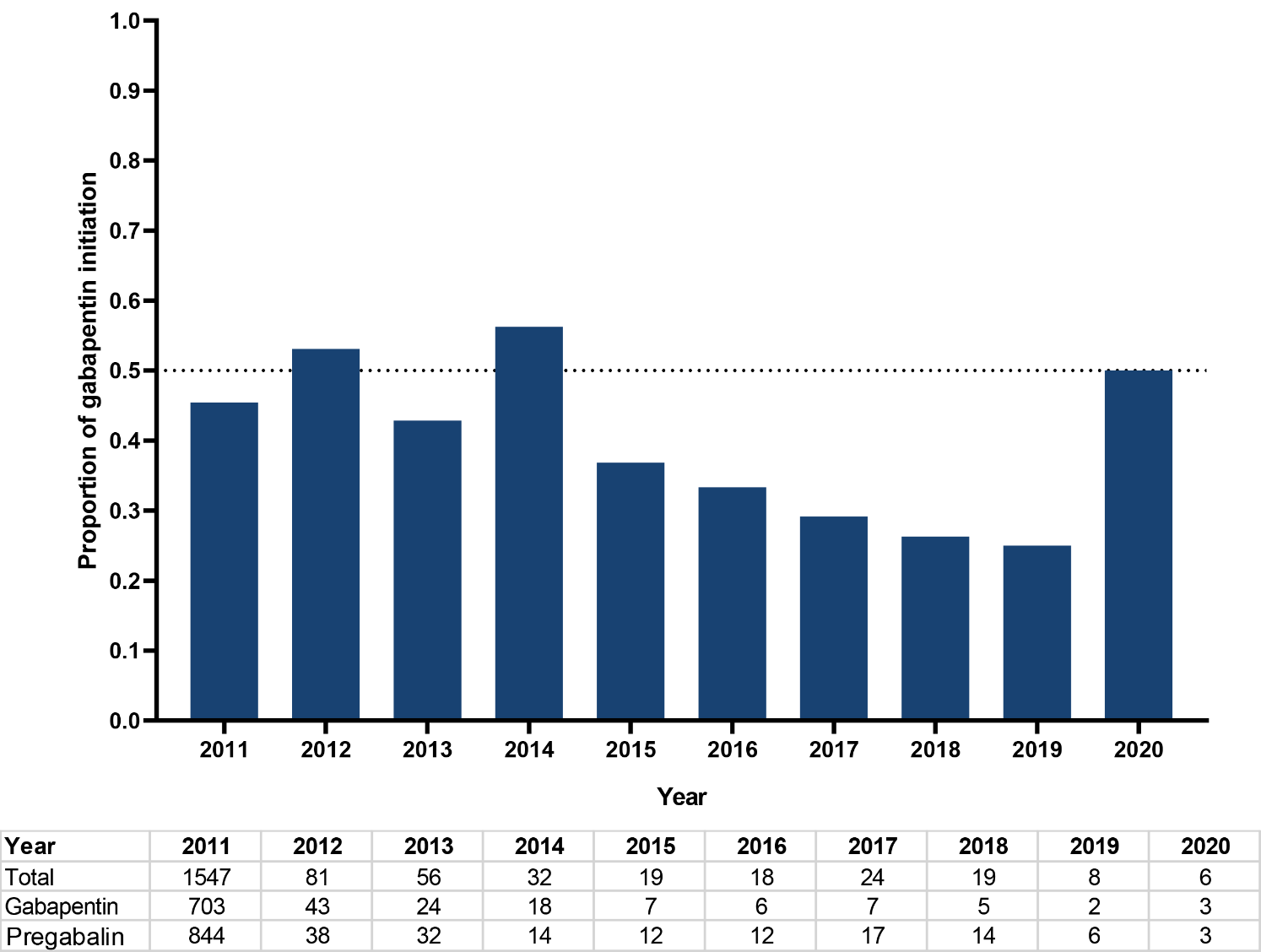
**

**Figure S1. The proportion of gabapentin use across years**

**
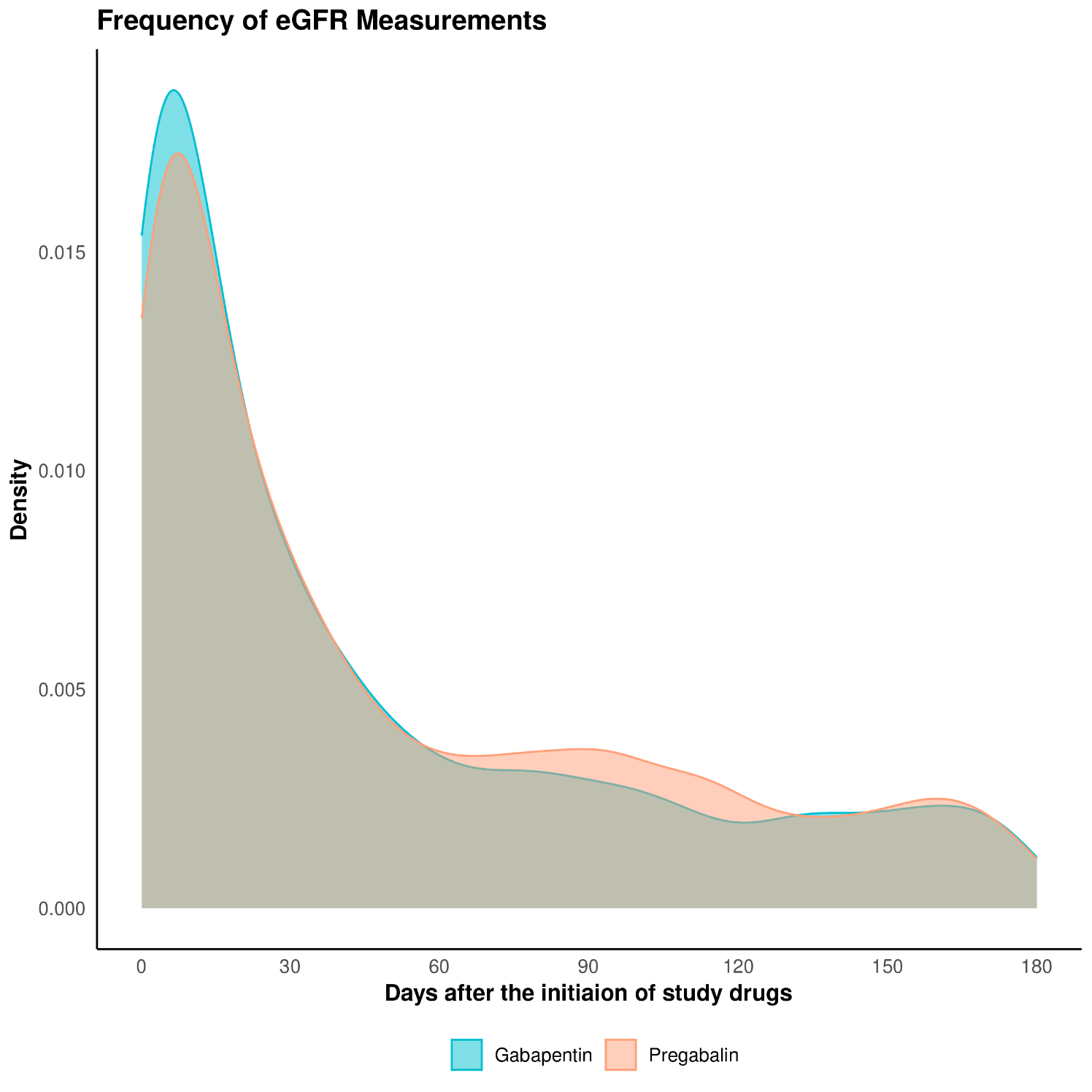
**

**Figure S2. The timings of the measurements stratified by gabapentinoids**

**Table S1. Specification and emulation of target trial**

| **Protocol component** | **Target trial specification** | **Target trial emulation** |
| --- | --- | --- |
| Eligible criteria | - Underwent an operation - No prior pregabalin or gabapentin use before the operation - No history of ESKD (eGFR< 15 ml/min/1.73 m^2^) on or before operation | Same as for the target trials |
| Treatment strategy | Initiate use of pregabalin or gabapentin at the perioperative period | Same as for the target trials. We defined the date of medication initiation to be the date of the first filled prescription. |
| Treatment assignment | Subjects are randomly assigned to a treatment strategy at baseline and will be aware of the treatment strategy they are assigned to. | Participants are assigned to the strategy compatible with their first filled prescription. The randomization was emulated by 1:1 propensity score matching |
| Outcomes | Primary outcome was kidney function decline (>40% decline in eGFR compared with the baseline).  Secondary outcomes included the incident CKD, AKI, and all-cause mortality. | Same as for the target trial |
| Follow-up | From index date until the occurrence of study outcomes, death, loss to follow-up or the administrative end of follow-up (31 December 2020) | Same as for the target trial |
| Causal contrast of interest | Intention-to-treat effect.  Per-protocol effect | Observational analog to intention-to-treat effect and the per-protocol effect (sensitivity analysis) |
| Statistical analysis | Intention-to-treat (ITT) analysis; Per-protocol analysis: censored the patients when they deviated treatment strategy. Subgroup analysis by age, sex, history of hypertension, diabetes, cancer, use of statins and PPI. | Same as for the target trial |

**Table S2. The proportion of missingness of covariates**

| **Covariates** | **Overall (%)** | **Gabapentin (%)** | **Pregabalin (%)** |
| --- | --- | --- | --- |
| BMI | 2.2 | 2.2 | 2.2 |
| SBP | 2.2 | 0.9 | 3.2 |
| DBP | 2.2 | 0.9 | 3.2 |
| Serum Albumin | 1 | 1.3 | 0.8 |
| Hemoglobin | 0.9 | 1 | 0.9 |

Abbreviations: DBP, diastolic blood pressure; SBP, systolic blood pressure

**Table S3. The association of kidney outcomes with the initiation of gabapentin versus pregabalin after propensity score overlap weighting**

| kidney outcomes | No. | Event | Person-year | Incidence rate (95%CI) ^*^ | HR (95%CI) |
| --- | --- | --- | --- | --- | --- |
| Kidney function decline |  |  |  |  |  |
| Pregabalin | 391 | 47 | 598 | 7.91(5.93-10.45) | 1.00(Reference.) |
| Gabapentin | 391 | 75 | 797 | 9.35(7.46-11.64) | 1.46(1.1-1.93) |
| Incident CKD^#^ |  |  |  |  |  |
| Pregabalin | 338 | 62 | 462 | 13.38(10.48-16.9) | 1.00(Reference.) |
| Gabapentin | 339 | 77 | 620 | 12.46(10.02-15.38) | 1.11(0.86-1.44) |

CKD, chronic kidney disease; HR, hazard ratio

^*^ per 100 person-year

^#^ Incident CKD was assessed among participants with baseline eGFR>60 ml/min/1.73m^2^

**Table S4. The association of kidney outcomes with the initiation of gabapentin versus pregabalin among participants with follow-up greater than 90 days**

| kidney outcomes | No. | Event | Person-year | Incidence rate (95%CI) ^*^ | HR (95%CI) |
| --- | --- | --- | --- | --- | --- |
| Kidney function decline |  |  |  |  |  |
| Pregabalin | 429 | 43 | 1013 | 4.25(3.13-5.73) | 1.00(Reference) |
| Gabapentin | 429 | 74 | 1272 | 5.82(4.62-7.29) | 1.54(1.04-2.27) |
| Incident CKD^#^ |  |  |  |  |  |
| Pregabalin | 330 | 45 | 754 | 5.97(4.44-7.97) | 1.00(Reference) |
| Gabapentin | 330 | 71 | 908 | 7.82(6.19-9.81) | 1.43(0.97-2.11) |

CKD, chronic kidney disease; HR, hazard ratio

^*^ per 100 person-year

^#^ Incident CKD was assessed among participants with baseline eGFR>60 ml/min/1.73m^2^

**Table S5. The association of kidney outcomes with the initiation of gabapentin versus pregabalin after excluding participants with AKI or death during the hospitalization.**

| kidney outcomes | No. | Event | Person-year | Incidence rate (95%CI) ^*^ | HR (95%CI) |
| --- | --- | --- | --- | --- | --- |
| Kidney function decline |  |  |  |  |  |
| Pregabalin | 594 | 52 | 952 | 5.46(4.14-7.15) | 1.00(Reference) |
| Gabapentin | 594 | 90 | 1211 | 7.43(6.05-9.09) | 1.52(1.08-2.14) |
| Incident CKD^#^ |  |  |  |  |  |
| Pregabalin | 525 | 83 | 706 | 11.76(9.52-14.42) | 1.00(Reference) |
| Gabapentin | 525 | 100 | 964 | 10.38(8.56-12.52) | 1.06(0.79-1.42) |

CKD, chronic kidney disease; HR, hazard ratio

^*^ per 100 person-year

^#^ Incident CKD was assessed among participants with baseline eGFR>60 ml/min/1.73m^2^

**Table S6. The association of kidney outcomes with the initiation of gabapentin versus pregabalin in per-protocol analysis**

| kidney outcomes | No. | Event | Person-year | Incidence rate (95%CI) ^*^ | HR (95%CI) |
| --- | --- | --- | --- | --- | --- |
| Kidney function decline |  |  |  |  |  |
| Pregabalin | 636 | 75 | 972 | 7.72(6.15-9.62) | 1.00(Reference) |
| Gabapentin | 636 | 109 | 1200 | 9.08(7.55-10.89) | 1.41(1.03-1.92) |
| Incident CKD^#^ |  |  |  |  |  |
| Pregabalin | 539 | 95 | 735 | 12.92(10.63-15.61) | 1.00(Reference) |
| Gabapentin | 539 | 116 | 954 | 12.16(10.19-14.45) | 1.10(0.82-1.47) |

CKD, chronic kidney disease; HR, hazard ratio

^*^ per 100 person-year

^#^ Incident CKD was assessed among participants with baseline eGFR>60 ml/min/1.73m^2^

**Table S7. The association of kidney outcomes with the initiation of gabapentin versus pregabalin confirmed by at least two consecutive measurements of eGFR**

| kidney outcomes | No. | Event | Person-year | Incidence rate (95%CI) ^*^ | HR (95%CI) |
| --- | --- | --- | --- | --- | --- |
| Kidney function decline |  |  |  |  |  |
| Pregabalin | 623 | 42 | 959 | 4.38(3.21-5.93) | 1.00(Reference) |
| Gabapentin | 623 | 87 | 1216 | 7.16(5.80-8.79) | 1.82(1.26-2.64) |
| Incident CKD^#^ |  |  |  |  |  |
| Pregabalin | 629 | 61 | 964 | 6.33(4.91-8.1) | 1.00(Reference) |
| Gabapentin | 629 | 93 | 1233 | 7.55(6.16-9.2) | 1.41(1.02-1.95) |

CKD, chronic kidney disease; HR, hazard ratio

^*^ per 100 person-year

^#^ Incident CKD was assessed among participants with baseline eGFR>60 ml/min/1.73m^2^

**Table S8. The association of kidney outcomes with the initiation of gabapentin versus pregabalin under the assumption of missing not at random**

| kidney outcomes | No. | Event | Person-year | Incidence rate (95%CI) ^*^ | HR (95%CI) |
| --- | --- | --- | --- | --- | --- |
| Kidney function decline |  |  |  |  |  |
| Pregabalin | 622 | 77 | 989 | 7.79(6.23-9.68) | 1.00(Reference) |
| Gabapentin | 622 | 125 | 1248 | 10.01(8.43-11.85) | 1.46(1.07-1.99) |
| Incident CKD^#^ |  |  |  |  |  |
| Pregabalin | 629 | 106 | 966 | 10.98(9.11-13.16) | 1.00(Reference) |
| Gabapentin | 629 | 149 | 1152 | 12.93(11.08-15.04) | 1.29(0.98-1.70) |

CKD, chronic kidney disease; HR, hazard ratio

^*^ per 100 person-year

^#^ Incident CKD was assessed among participants with baseline eGFR>60 ml/min/1.73m^2^
